# Does the number of doctors on call correlate with patient admission rates and patient morbidity at Danish Medical Admissions Units?

**DOI:** 10.1186/1757-7241-20-S2-P15

**Published:** 2012-04-16

**Authors:** Lars Folkestad, Peter Hallas, Mikkel Brabrand, Hasse Blom

**Affiliations:** 1 Danish Society for Emergency Medicine, Denmark; 2 Intensivafsnittet 0531, Nordsjælands Hospital, Hilleroed, Denmark

## Background

The morbidity of patients admitted throughout the day and night may vary, but it is unknown whether the physician staffing vary in accordance with variations in caseload, and patient complexity.

## Methods

This descriptive study use a mixture of methods. Data on staffing were retrieved from a cross-sectional study amongst all Danish MAUs. Data regarding admissions rates were retrieved from Sydvestjysk Sygehus (SVS) in Esbjerg registry. Modified Early Warning Score (MEWS) was calculated on all patients admitted to the MAU at SVS over three months. Data regarding admissions rats to intensive care unit (ICU) were collected from Hillerød Hospital. Data will be presented descriptively. X2-test and linear regression was used for analysis.

## Results

98% of all MAUs have a junior house officer (JHO) and 36% have a senior house officer (SHO), on call round the clock. All MAUs have a specialist registrar (SR) on call from 8 a.m. until 8 p.m. Seventy nine % of all admissions occur between 8 a.m. and 8 p.m. On average 34 % of the patients have more than two abnormal vital signs using the MEWS scale with no significant differences comparing the proportion of abnormal vital signs from 8 a.m.-8 p.m., 8 p.m.-11 p.m. and 11 p.m.-8 a.m., p = 0.44. Approximately 70 % of all admissions to the ICU occurs between 8 a.m. and 8 p.m., 10% from 8 p.m. to 11 p.m. and 20% from 11 p.m. to 08 a.m., p < 0.001, where all patients are admitted by a specialist registrar. There is a significant correlation between the proportion of JHO on call and the proportion of admissions to MAU (r = 0.80) and the ICU (r = 0.93), and a significant correlation between the proportion of SR on call and proportion of admissions to MAU (r = 0.86) and the ICU (r = 0.91). However, there is no significant correlation between the proportion of SR on call and the proportion of abnormal vitals signs (r = -0.23).

## Conclusion

At the Danish MAUs, the staffing correlates to the patient admissions rate. However, staffing experience does not correlate with case complexity in on-call hours.

